# Brazilian version of “The Insulin Delivery System Rating Questionnaire”: translation, cross-cultural adaptation and validation

**DOI:** 10.20945/2359-3997000000274

**Published:** 2020-07-17

**Authors:** Raquel Cristina Lopes Assis Coelho, Adriana Silvina Pagano, Aleida Nazareth Soares, Janice Sepulveda Reis

**Affiliations:** 1 Santa Casa de Belo Horizonte Belo Horizonte MG Brasil Ensino e Pesquisa, Santa Casa de Belo Horizonte, Belo Horizonte, MG, Brasil; 2 Faculdade de Letras Universidade Federal de Minas Gerais Belo Horizonte MG Brasil Faculdade de Letras, Universidade Federal de Minas Gerais, Belo Horizonte, MG, Brasil

**Keywords:** Translation, cultural adaptation, diabetes mellitus, insulin, health related quality of life

## Abstract

**Objective:**

The aim of this study was to translate and cross-culturally adapt the Insulin Delivery System Rating Questionnaire (IDSRQ) for Brazilian users. Validation and reliability analysis of measures were also performed.

**Materials and methods:**

Methodological study comprising the following stages: forward translation, synthesis, back-translation, assessment by Expert Committee, pre-test and validation. International guidelines for translation and cross-cultural adaptation of measurement tools were followed. Validation data provided information about reliability (internal consistency, test-retest) and construct validity of the IDSRQ.

**Results:**

Content validation by Experts’ assessment was successful, with a mean Content Validity Index of 0.87 (±0.2). The IDSRQ validation study involved 113 T1DM patients, 46% male, mean age 32.61 (±12.59) years and mean age at diagnosis of diabetes of 17.51 (±12.41). The scale presented good internal consistency (Cronbach’s alpha =0.786). The reliability analysis of the instrument was conducted by calculating the Intra-class Correlation Coefficient 0.885 (0.834-0.921), which indicated adequate concordance in all measures.

**Conclusion:**

The translated and cross-culturally adapted Brazilian Portuguese version of the IDSRQ may be used to assess health-related quality of life (HRQOL) and treatment preferences for insulin delivery systems in T1DM Brazilian patients.

## INTRODUCTION

In the treatment of patients with chronic non-communicable diseases, health care professionals should take into account both objective factors (clinical and socio-demographic data) and subjective factors, such as sense of satisfaction with current and previous treatments ( 1 , 2 ).

The World Health Organization (WHO) defines quality of life as perceived by the person’s own life situation within his/her cultural context and value system and in relation to his/her objectives and interests ( 3 ). This aspect is particularly important in the case of diabetes mellitus (DM), where treatment requires the patient’s major commitment, often with adjustments of his/her current lifestyle to requirements of the treatment ( 3 , 4 ).

Some diabetes-specific quality of life measures have been developed ( 5 , 6 ). Most of them were originally developed for use among English-speaking patients. The Insulin Delivery System Rating Questionnaire (IDSRQ) was designed to assess the impact of different insulin delivery systems as comprehensively as possible ( 6 ). It includes measures that are both general (overall quality of life) and diabetes specific (perceived clinical efficacy, treatment satisfaction and burden, and diabetes-related worries and social burdens) ( 6 ).

The IDSRQ provides a multidimensional measure of overall preference, incorporating not only a retrospective comparison with the previously used delivery system but also a prospective measure of interest in changing current system. It is designed to be used with any insulin delivery system and to permit comparisons of various systems (e.g., pen versus syringe, one pump versus another pump etc.) An instrument like the IDSRQ is not available in Brazil and might be useful to improve diabetes care ( 7 ).

The aim of this study was to translate and culturally adapt the IDSRQ into Brazilian Portuguese. We also tested the psychometric properties of the translated version in a Brazilian validation sample.

## MATERIALS AND METHODS

### Translation and cultural adaptation

The IDSRQ was originally created and is available in English and has not been used in Brazil so far. The author of the measure consented to its translation and application in Brazilian Portuguese.

This methodological study was performed in accordance with generally accepted international principles of translation and cross-cultural adaptation of measurement tools ( 8 ). The translation process included the following steps: preparation; forward translation; back translation; back translation review; proofreading and final report ( 9 ).

Translation from English into Portuguese was performed by two independent translators, graduate students in Translation Studies, whose mother tongue was Brazilian Portuguese (forward translation). An analysis and comparison of the translations was subsequently performed and a consensus version of the questionnaire in Portuguese was reached. The next step was for two other independent translators to re-translate the consensus version of the tool from Portuguese back into English (backward translation).

**Figure 1:**
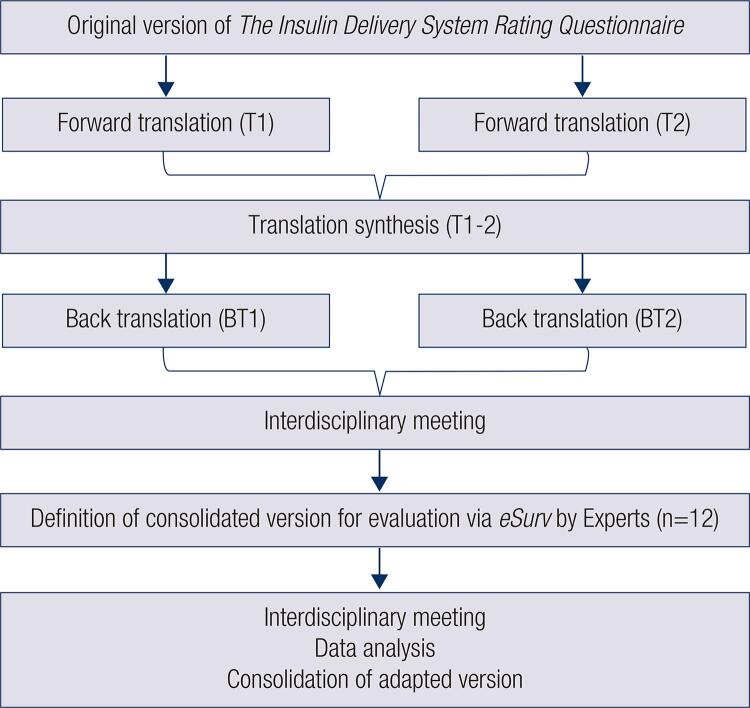
Translation and cross-cultural adaptation steps

The two back-translated versions were compared with the original text, and no significant differences were found. After these steps, 10 healthcare professionals (physicians, nurses and dieticians) and 8 applied linguists were invited to participate as an Expert Committee. Figure 1 summarizes translation and cultural adaptation steps.

### Expert committee analysis

Members of the Expert Committee were selected based on their expertise in diabetes and English proficiency, assessed through their curriculum vitae (www.plataformalattes.com.br). The invitation was sent by e-mail and a link provided for access to the instrument previously uploaded to the web *e-Surv* platform. The experts were divided into two groups so that assessment would not take longer than 60 minutes. All the participants assessed the translated instructions of the instrument as well as the scales for respondents to record their answers so that they would assess the translated version in its entirety as prospective respondents would receive it and fill it in. The aim was to evaluate semantic, idiomatic, conceptual and experiential equivalence between the original and translate items. The experts were asked to evaluate each statement for ease of understanding and clarity of the information and to present suggestions for improvement of the text.

When comparing the original and the translated version, the experts assessed the translated instrument in terms of need for retranslation (1 = requires complete retranslation; 2 = requires partial retranslation with substantial editing; 3 = requires partial retranslation with minor editing to improve the text; 4 = does not require retranslation). After obtaining the experts’ responses, the Content Validity Index (CVI) was calculated, defined by the sum of the relative frequencies of “3” and “4” responses. The assumption was that the higher the CVI, the lower the amount of editing needed to improve the text ( 10 ).

### Validity and reliability

Assessment of the reliability and validity of the Brazilian Portuguese IDSRQ was carried out in a sample of Brazilian patients with T1DM who were using continuous subcutaneous insulin infusion (CSII) or multiple daily injections (MDI) of insulin.

To calculate the sample size, a psychometric property was chosen that involves both the moment of the test and of the retest, the temporal reproducibility, and an alternative to its measure, the linear correlation. Thus, a significance level of 5%, test power of 80%, standard deviation equal in the test and retest scores and a correlation coefficient of 0.30 (minimum value to be detected in the evaluation of reliability) were considered. The minimum sample size required was 80 individuals. When considering a 20% loss, the final sample size required was 100 individuals.

### Validation subjects

Participants were recruited among patients with T1DM using MDI or CSII in ambulatory settings at the Department of Endocrinology, at Hospital Santa Casa de Belo Horizonte (public health system). Further recruitment was done at the practices of two physicians in the same city. Patients were contacted by the researcher (Coelho RCLA) and asked to participate in the study. Those who expressed interest received the link to the questionnaire via e-mail. At the follow up, a new link to the questionnaire was sent to the provided e-mail address 2-3 weeks after receiving the initial questionnaire. The researcher ensures with patients that there were no significant clinical changes in the interval. General inclusion criteria were T1DM for ≥ 6 months; treatment with insulin in CSII or MDI (three or more injections/day); patient’s agreement consent and physical and psychological health condition allowing independent completion of questionnaire; and age above 10 years old.

The study was approved by the Research Ethics Committee of Santa Casa De Belo Horizonte Group (CAAE number 65656117.6.1001.5138). The consent form was made available electronically on the first page of the web questionnaire, where the patients recorded their agreement to participate in the study.

### Statistical analysis

Descriptive analysis of the categorical variables was performed by calculating absolute and relative frequencies, whereas for quantitative variables, the means, standard deviation, and percentiles were calculated.

The score for each item was a metric ranging from 0 for the lowest response option to 100 for the highest response option, with equal distance between response categories. Scale scores were computed as the mean of the completed items ( 6 ).

The evaluation of internal consistency was made from the calculation of Cronbach’s alpha. Cronbach’s alpha is computed by correlating the score for each scale item with the total score for each observation and then comparing that to the variance for all individual item scores.

Test-retest analysis used the intraclass correlation coefficient (ICC) and Student’s *t* tests of mean differences. ICC was calculated in a two-way mixed effects model where people effects are random and measures effects are fixed.

Floor and ceiling effects were measured by the number of respondents receiving the minimum and maximum scores, respectively.

Data analysis was carried out using the SPSS statistical software, version 23. The significance level considered for the statistical tests was 5%.

## RESULTS

### Expert Committee

Out of the 18 experts invited to participate in the Expert Committee, 12 completed questionnaires were obtained, 8 of them by healthcare professionals and 4 by applied linguists. 75% of the experts reported having completed graduate studies (master and doctoral levels) ( Table 1 ).

**Table 1:** Characteristics of participants in Expert Committee (n = 12)

Variables	n (%)*
Male/female	5 (41.66%) / 7 (58.34%)
Age (years)	
20-30	3 (25%)
31-40	7 (58.34%)
41-50	2 (16.66%)
Domain	
Medicine	4 (33.33%)
Nursing	2 (16.67%)
Nutrition	2 (16.67%)
Languages	4 (33.33%)
Graduate studies	
Diploma course	3 (25%)
Master’s degree	5 (41.66%)
Doctoral degree	4 (33.33%)

*n (%): absolute and relative frequencies.

### Content validity index (CVI)

In general, the instrument presented reasonable levels of CVI, resulting in a mean CVI of 0.87 (±0.2). Table 2 shows absolute and relative frequencies of the responses by Expert Committee participants in the evaluation of the instrument items and content validity index.

**Table 2:** Absolute and relative frequencies of Expert Committee responses in the evaluation of the instrument items and content validity index

Item*	Requires complete retranslation	Requires partial retranslation with substantial editing	Requires partial retranslation with minor editing	Does not require retranslation	CVI
1			4 (57.14%)	3 (42.86%)	1.00
2			1 (14.28%)	6 (85.72%)	1.00
3		2 (33.33%)	3 (50.00%)	1 (16.67%)	0.66
4	1 (16.67%)	1 (16.67%)	3 (50.00%)	1 (16.67%)	0.66
5		1 (16.67%)	3 (50.00%)	2 (33.33%)	0.84
6			1 (16.67%)	5 (83.33%)	1.00
7			3 (60.00%)	2 (40.00%)	1.00
8			3 (60.00%)	2 (40.00%)	1.00
9			3 (60.00%)	2 (40.00%)	1.00
10		3 (60.00%)	1 (20.00%)	1 (20.00%)	0.40
11			3 (75.00%)	1 (25.00%)	1.00
				Mean CVI	0.87
				SD	0.20

CVI: Content Validity Index; SD: Standard Deviation.

*1: patient characteristics; 2: frequency of blood glucose monitoring; 3: insulin delivery system current using; 4: treatment satisfaction; 5: daily activity interference; 6: glucose monitoring; 7: clinical efficacy; 8: diabetes worries; 9: diabetes burden; 10: psychological well-being; 11: overall preference.

Editing was performed on the translated version following the suggestions by the experts regarding:

- Narrow meaning of terms – the item “If you take injections, how many injections do you take per day?”, had been initially translated as: [If you use insulin, how many insulin injections do you take per day?]. The experts argued that the proposed rendition could be narrowly interpreted as referring to injectable insulin. As alternative forms of insulin administration are known, such as inhaled insulin, aiming at longer usability of the translated instrument and less need for updates, we opted for the broader term [insulin administration] throughout the questionnaire, which includes the narrower term subcutaneous insulin injections.
- Type of question - the item “ *How satisfied are you with your current insulin delivery system* ?” had been initially translated as *[Are you satisfied with your current insulin delivery system?],* as the original “how satisfied” poses a problem in Portuguese since this kind of question is less frequent than in English. The experts argued a “yes – no” question could end up eliciting a binary answer. To solve that problem, an introductory phrase was added to the translated item: *[Regarding your current insulin delivery system...].*

Discussion was prompted by the translation of the item “ *Uncertainty about getting the amount of insulin intended* ” initially translated as [Uncertainty about the amount of insulin that has to be administered”]. The experts suggested two alternative renditions: [Uncertainty about delivery of the amount of insulin intended], [Uncertainty about the amount of insulin that needs to be administered]. After discussing these suggestions among healthcare professionals and linguists, we opted for *[Uncertainty whether the system administers the amount of insulin you need to take].*

### Validation sample characteristics

A total of 113 individuals with T1DM participated in the validation step. Table 3 shows their sociodemographic data. The majority of participants (61.1%) reported to be using pens as insulin delivery system at the time of questionnaire filling. The sample was primarily with T1DM patients (100%), women (54%) and individuals with mean age of 32.61 years old. Most subjects (88.5%) reported monitoring their blood glucose three or more times per day.

**Table 3:** Demographic and clinical characteristics of individuals with T1DM who participated in the validation step (n=113)

Variables	n (%)*	
Age (years)	32.61 (±12.59)	
Sex		
Male	52 (46%)	
Female	61 (54%)	
Mean age at diagnosis of diabetes (years)	17.51 (±12.41)	
Education		
Unfinished elementary school	13 (11%)	
Finished elementary school	39 (35%)	
Unfinished high school	35 (31%)	
Finished high school	16 (14%)	
College	10 (9%)	
Current insulin delivery system		
Vial and syringe	31 (27.4%)	
Pen	69 (61.1%)	
Insulin pump	13 (11.5%)	
Number of insulin injections taken per day		
3		21 (18.6%)
4		20 (17.7%)
5		26 (23%)
6		20 (17.7%)
7		7 (6.2%)
>7		19 (16.8%)
Current insulin		
NPH		45 (39.8%)
Glargine U100		39 (34.5%)
Other basal insulin		29 (25.7%)
Lispro		68 (62.4%)
Asparte		25 (22.9%)
Glulisine		6 (5.6%)
Regular		10 (9.1%)
Frequency of blood glucose monitoring		
Less than twice daily		5 (4.4%)
Twice daily		8 (7.1%)
Three times daily		31 (27.4%)
More than three times daily		69 (61.1%)

* n (%): Absolute and relative frequencies. “Age” and “mean age at diagnosis of diabetes” are means ± SD.

### Validity and reliability analysis

Table 4 shows the validity and reliability evaluation for the Brazilian IDSRQ measures. The overall Cronbach’s alpha value was 0.786, indicating good internal consistency. Chronbach’s alpha coefficients for the Brazilian version ranged from 0.697-0.906 for the following measures: treatment satisfaction, daily clinical interference, clinical efficacy, diabetes worries and social burden. Internal consistency of the measure psychological well-being was 0.288. Cronbach’s alpha if psychological well-being measure is removed was 0.810 (0.756-0.856).

**Table 4:** Validity and reliability evaluation of the Brazilian IDSRQ measures

Measure (no. items)	Initial mean	Actual range (1-100)	Floor (%)	Ceiling (%)	Internal consistency (Cronbach’s Alpha) α	Cronbach’s Alpha if measure is removed (95% CI for Cronbach’s Alpha)	Intraclass Correlation Coefficient ICC (95% CI)	Mean response shift
Treatment satisfaction (15)	66.52 (±21,15)	13.3-100	0	3	0.901	0.789 (0.726-0.843)	0.972 (0.958-0.982)	1.68
Daily activity interference (11)	31.54 (±25.10)	0-100	8	0	0.906	0.863 (0.823-0.899)	0.956 (0.933-0.971)	-2.61*
Clinical efficacy (9)	76.63 (±15.47)	30.5-100	0	7	0.843	0.795 (0.735-0.848)	0.941 (0.902-0.965)	2.94*
Diabetes worries (6)	60.68 (±19.67)	12.5-100	0	4	0.796	0.833 (0.784-0.876)	0.958 (0.936-0.972)	-0.89
Social burden (7)	50 (±17.84)	7.4-100	0	1	0.697	0.831 (0.780-0.874)	0.942 (0.912-0.962)	1.05
Psychological well-being (15)	57.16 (±13.53)	28.3-86.7	0	1	0.288	0.810 (0.756-0.856)	0.971 (0.956-0.981)	2.74*
General score		0-100	2	20	0.786		0.885 (0.834-0.921)	

Data are means ± SD, unless otherwise indicated. *P ≤ 0.05. CI: confidence interval.

Out of the 113 participants who completed the initial questionnaire, 92 (81.4%) completed the follow-up questionnaire and were used in the test-retest analysis. Test-retest reliability ranged from 0.941-0.972 and overall ICC was 0.885 (0.834-0.921).

Floor effects (percent with minimum score) ranged from 0 to 8% (median 0%), and ceiling effects (percent with maximum score) ranged from 0 to 20% (median 3%).

A copy of the instrument is available in Supplement Material .

## DISCUSSION

The results of our study showed the reliability and validity of the IDSRQ in its Brazilian version after cross-cultural adaptation and validation in T1DM patients. The decision to cross-culturally adapt an instrument has to do with the various advantages reported in the literature, such as saving time and the possibility of comparing the results with studies carried out in different countries ( 11 ).

Although there is no gold standard template to follow for translation and cross-cultural adaptation, four steps are essential and are reported in guidelines and recommendations: translation, back-translation, review by an expert committee and pretesting ( 12 ). All steps were rigorously followed in this study to preserve social, cultural and linguistic characteristics.

Regarding cross-cultural adaptation, CVI of 0.78 or more is expected for translated instruments ( 13 , 14 ). Accordingly, we obtained a CVI of 0.87.

Sample characteristics in this study were different from the original IDSRQ validation study. In the original validation study, 72.1% of the sample was T1DM patients ( 6 ). Our sample was 100% T1DM patients. Also, our study had 11.5% of the patients using CSII versus 71.1% in Peyrot and Rubin’s study. Two major strengths of the study were fulfillment of the sample suggested by the sample calculation and high rate of patients answering the retest (81.4%) versus 57.8% in the study conducted by Peyrot & Rubin ( 6 ). The absence of T2DM patients is a limitation of our study. We opted for not including T2DM patients in the validation study because most T2DM patients interviewed in the pre-test phase would need assistance to fill the questionnaire.

Cronbach’s alpha was 0.78, which indicates good internal consistency. Regarding psychometric properties, the α reliability coefficients ranged from 0.67 to 0.92 in the study conducted by Peyrot and Rubin ( 6 ). The α reliability coefficients ranged from 0.288-0.906 in our study. The smaller α was obtained in *psychological well-being* measure. Similarly, in the validation study of IDSRQ for Italian ( 15 ), the smaller α was also in this scale. In turn, even if the *psychological well-being* measure were excluded, there would be a slight change in Cronbach’s alpha, as shown in Table 4 . For this reason, associated with the importance of the measure, the authors have opted for retaining this measure.

Test-retest reliability was performed in a two-week interval. ICC values ranged from 0.941-0.972 in our study, and from 0.67-0.94 in the original study. Also, our study showed small shifts in the means scores over time. Both results indicate good reliability of the translated version.

Assessment of patient-reported outcomes, especially treatment satisfaction, is increasingly recognized as important in determining the efficacy of new therapies ( 16 ). Treatment satisfaction may be associated with adherence to treatment, glycemic control, and treatment preference. Healthcare professionals need validated tools to evaluate treatment preferences by patients with diabetes. IDSRQ is a reliable tool and already used in other studies ( 17 - 19 ). An instrument like IDSQR will be useful for Brazilian clinicians and researchers.

In conclusion, the Brazilian version of IDSRQ was translated, cross-culturally adapted and validated in T1DM patients. It is a promising and useful tool to clinicians and researchers to assess patient perception of their insulin delivery systems. Given the importance of insulin in the management of diabetes and increasingly available alternatives insulin delivery systems, we believe that the application of the instrument may contribute to implementing care practices based on patients’ preferences.

## References

[1] Bhatt JK, Thomas S, Nanjan MJ. Health Outcome Measures for Diabetes Mellitus: A Review. Appl Res Qual Life. 2012;7:413-43.

[2] Dickinson JK, Guzman SJ, Maryniuk MD, O’Brian CA, Kadohiro JK, Jackson RA, et al. The use of language in diabetes care and education. Diabetes Care. 2017;40(12):1790-9. 10.2337/dci17-0041 29042412

[3] Haas L, Maryniuk M, Beck J, Cox CE, Duker P, Edwards L, et al. National standards for diabetes self-management education and support. Diabetes Care. 2013;36 Suppl 1(Suppl 1):S100-8. 10.2337/dc13-S100 PMC3537270 23264420

[4] Matza LS, Stewart KD, Paczkowski R, Coyne KS, Currie B, Boye KS. Psychometric evaluation of the Diabetes Injection Device Experience Questionnaire (DID-EQ) and Diabetes Injection Device Preference Questionnaire (DID-PQ). J Patient Rep Outcomes. 2018;2:44. 10.1186/s41687-018-0064-3 PMC6153201 30294714

[5] Peyrot M, Xu Y, Rubin RR. Development and validation of the diabetes medication system rating questionnaire-short form. Diabet Med. 2014;31(10):1237‐44. 10.1111/dme.12453 PMC4232890 24673614

[6] Peyrot M, Rubin RR. Validity and Reliability of an Instrument for Assessing Health-Related Quality of Life and Treatment Preferences The Insulin Delivery System Rating Questionnaire. Diabetes Care. 2005;28(1):53-8. 10.2337/diacare.28.1.53 15616233

[7] Gomes MB, Cobas RA, Matheus AS, Tannus LR, Negrato CA, Rodacki M, et al. Regional differences in clinical care among patients with type 1 diabetes in Brazil: Brazilian Type 1 Diabetes Study Group. Diabetol Metab Syndr. 2012;4(1):44. 10.1186/1758-5996-4-44 PMC3538646 23107314

[8] Santo RM, Ribeiro-Ferreira F, Alves MR, Epstein J, Novaes P. Enhancing the Cross-Cultural Adaptation and Validation Process: Linguistic and Psychometric Testing of the Brazilian-Portuguese Version of a Self-Report Measure for Dry Eye. J Clin Epidemiol. 2015;68(4):370-8. 10.1016/j.jclinepi.2014.07.009 25619561

[9] Landim CAP. Adaptação cultural para o Brasil e Portugal do instrumento Patient Assessment of Chronic Illness Care (PACIC); 2012.

[10] Maindal HT, Kayser L, Norgaard O, Bo A, Elsworth GR, Osborne RH. Cultural adaptation and validation of the Health Literacy Questionnaire (HLQ): robust nine-dimension Danish language confirmatory factor model. Springerplus. 2016;5(1):1232. 10.1186/s40064-016-2887-9 PMC4971008 27536516

[11] Vieira G de LC, Pagano AS, Reis IA, Rodrigues JSN, Torres H de C. Translation, cultural adaptation and validation of the Diabetes Attitudes Scale - third version into Brazilian Portuguese. Rev Lat Am Enfermagem. 2018;25:e2875. 10.1590/1518-8345.1404.2875 PMC5768205 29319739

[12] De Souza Bastos VC, Carneiro AAL, Ramos Barbosa M dos S, De Andrade LB. Brazilian version of the Pediatric Functional Status Scale: Translation and cross-cultural adaptation. Rev Bras Ter Intensiva. 2018;30(3):301-7. 10.5935/0103-507X.20180043 PMC6180463 30183976

[13] Beaton DE, Bombardier C, Guillemin F, Ferraz MB. Guidelines for the process of cross-cultural adaptation of self-report measures. Spine (Phila Pa 1976). 2000;25(24):3186-91. 10.1097/00007632-200012150-00014 11124735

[14] Coluci MZO, Alexandre NMC, Milani D. Construção de instrumentos de medida na área da saúde. Ciênc Saúde Coletiva. 2015;20(3):925-36. 10.1590/1413-81232015203.04332013 25760132

[15] Cherubini V, Gesuita R, Bonfanti R, Franzese A, Frongia AP, Iafusco D, et al. Health-related quality of life and treatment preferences in adolescents with type 1 diabetes. The VIPKIDS study. Acta Diabetol. 2014;51(1):43-51. 10.1007/s00592-013-0466-x 23508374

[16] Peyrot M, Rubin RR. How does treatment satisfaction work? Modeling determinants of treatment satisfaction and preference. Diabetes Care. 2009;32(8):1411-7. 10.2337/dc08-2256 PMC2713611 19470837

[17] Alsairafi ZK, Smith FJ, Taylor KMG, Alsaleh F, Alattar AT. A qualitative study exploring patients’ experiences regarding insulin pump use. Saudi Pharm J. 2018;26(4):487-95. 10.1016/j.jsps.2018.02.010 PMC5962644 29844719

[18] Bergenstal RM, Peyrot M, Dreon DM, Aroda VR, Bailey TS, Brazg RL, et al. Implementation of Basal-Bolus Therapy in Type 2 Diabetes: A Randomized Controlled Trial Comparing Bolus Insulin Delivery Using an Insulin Patch with an Insulin Pen. Diabetes Technol Ther. 2019;21(5):273-85. 10.1089/dia.2018.0298 PMC6532545 31025878

[19] Peyrot M, Dreon D, Zraick V, Cross B, Tan MH. Patient Perceptions and Preferences for a Mealtime Insulin Delivery Patch. Diabetes Ther. 2018;9(1):297-307. 10.1007/s13300-017-0365-1 PMC5801251 29327220

